# First fatal human bloodstream infection caused by *Macrococcus caseolyticus* subsp. *caseolyticus* in China: genomic insights into virulence and antimicrobial resistance

**DOI:** 10.3389/fcimb.2026.1825695

**Published:** 2026-06-01

**Authors:** Lifeng Shi, Yafei Ruan, Xiuling Wang, Jia Xu, Ji Pu, Yingmiao Zhang, Zhongxin Lu

**Affiliations:** 1 Department of Medical Laboratory, The Central Hospital of Wuhan, Tongji Medical College, Huazhong University of Science and Technology, Wuhan, China; 2 School of Laboratory Medicine, Hubei University of Chinese Medicine, Wuhan, China; 3 National Key Laboratory of Intelligent Tracking and Forecasting for Infectious Diseases, National Institute for Communicable Disease Control and Prevention, Chinese Center for Disease Control and Prevention, Beijing, China

**Keywords:** antimicrobial resistance, biofilms, *M. caseolyticus* subsp. *caseolyticus*, MALDI-TOF MS, virulence factors, whole genome sequencing

## Abstract

**Background:**

*Macrococcus caseolyticus* is a Gram-positive bacterium mainly isolated from dairy products and animal skin and is usually associated with animal infections. *M. caseolyticus* comprises two subspecies, *M. caseolyticus* subsp. *caseolyticus* and *M. caseolyticus* subsp. *hominis*. Here, we report a fatal bloodstream infection caused by *M. caseolyticus* subsp. *caseolyticus* in China and characterize the clinical isolate by genomic and phenotypic analyses.

**Methods:**

Strain WH712 was isolated from the blood culture of a 91-year-old male patient and identified by matrix-assisted laser desorption ionization/time-of-flight mass spectrometry. Whole-genome sequencing was performed using the Illumina HiSeq platform. Antimicrobial susceptibility testing was conducted using the AutoMic-i600 system, and biofilm-forming capacity was evaluated using a 96-well microtiter plate assay.

**Results:**

WH712 was initially identified as *M. caseolyticus* by MALDI-TOF MS and confirmed as *M. caseolyticus* subsp. *caseolyticus* by whole-genome analysis. Homology-based genomic analysis identified putative homologs of *bopD*, *ami*, *srtE*, and *pfbA*, which have been associated in other bacteria with biofilm-related regulation, adhesion, and cell-surface functions. These findings suggest that WH712 may harbor genomic features relevant to host colonization and persistence. The isolate was phenotypically susceptible to most tested antibiotics but showed intermediate resistance to clindamycin, possibly associated with a *vgaB* homolog and efflux-related homologs, including *macB* and *sdrM*. Phenotypic testing demonstrated that WH712 had strong biofilm-forming capacity, comparable to that of *Staphylococcus aureus* ATCC 29213, which may have contributed to bacterial persistence and the poor clinical outcome.

**Conclusions:**

This study reports a fatal human bloodstream infection associated with *M. caseolyticus* subsp. *caseolyticus* in China. Whole-genome analysis and phenotypic testing showed that this clinical isolate had a strong biofilm-forming phenotype, which may have contributed to bacterial persistence. However, the poor clinical outcome should be interpreted primarily in the context of the patient’s advanced age, severe comorbidities, and impaired host defenses.

## Introduction

The genus *Macrococcus* comprises Gram-positive cocci and was first proposed in 1998 (Kloos et al., 1998). It belongs to the *Staphylococcaceae* family and has often been misclassified as the *Staphylococcus* genus. However, it was differentiated from *Staphylococcus* based on ribotype pattern types, DNA–DNA hybridization, and phenotypic properties (Kloos et al., 1998; Mazhar et al., 2018). According to the List of Prokaryotic names with Standing in Nomenclature (LPSN) database (https://lpsn.dsmz.de/genus/*Macrococcus*>), *Macrococcus* currently has 16 species. The genus is commonly found on animal skin surfaces and meat products, indicating its commensal relationship with animals. *Macrococcus* species can cause various animal infections, such as otitis, rhinitis, mastitis, and necrotizing fasciitis (de la Fuente et al., 1992; Cotting et al., 2017; Schwendener et al., 2017; Acheampong et al., 2021). Among the 16 recognized species, only a few of them have been reported to be associated with human clinical specimens or infectious diseases, including *M. canis* (Jost et al., 2021), *M. bohemicus*, *M. goetzii*, *M. epidermidis* (Mašlaňová et al., 2018), and *M. psychrotolerans*. *M. caseolyticus* is a member of the genus *Macrococcus*, which comprises two subspecies: *M. caseolyticus* subsp. *hominis* and *M. caseolyticus* subsp. *caseolyticus*. *M. caseolyticus* subsp. *hominis* has been isolated from humans (Mašlaňová et al., 2018), and a methicillin-resistant human isolate of *M. caseolyticus* (strain 26J7727, Isolate ID: 21) was documented in the pubMLST database (Jolley et al., 2018). No relevant clinical cases of human infection have ever been reported for *M. caseolyticus* subsp. *caseolyticus*. Previous studies on this type of bacteria mainly focused on issues related to veterinary medicine and food safety. A strain of *M. caseolyticus* causing high mortality rates in commercial broiler chickens was reported in China (Li et al., 2018). However, no cases of life-threatening *M. caseolyticus* infections have been reported in humans. Currently, most of the available WGS data related to *M. caseolyticus* is derived from meat products (Baba et al., 2009; Li et al., 2018; Macfadyen et al., 2018; Zhang et al., 2022). The potential pathogenicity of *M. caseolyticus* to humans is largely unknown. Therefore, the objective of this study was to comprehensively investigate the first fatal human case of bacteremia caused by *M. caseolyticus* subsp. *caseolyticus*. By utilizing WGS and phenotypic assays, we aimed to characterize its antimicrobial resistance profile, virulence factors, and biofilm-forming capacity, thereby elucidating the potential pathogenic mechanisms of this emerging zoonotic threat.

## Materials and methods

### Bacterial strain isolation and MALDI-TOF MS identification

The positive blood culture specimens were inoculated on MacConkey agar and Columbia blood agar plates (Guangzhou Dijing Microbial Technology Co., Ltd., Guangzhou, China). The plates were then incubated overnight at 35°C in the presence of 5% CO_2_. Additionally, a blood film was prepared from the blood culture bottle for microscopic examination using Gram staining. The strains isolated from blood cultures were identified using MALDI-TOF MS. Single bacterial colonies were directly spotted on a target plate and analyzed using the VITEK MS (bioMérieux, France) following the manufacturer’s protocols. The mass spectra obtained from the tested isolate were compared with the standard reference spectra in the VITEK MS 3.2 database to identify the species.

### Genome sequencing, assembly, and annotation

Whole-genome sequencing of strain WH712 was performed using the Illumina HiSeq platform by Sangon Biotech Co., Ltd. (Shanghai, China). The raw reads were cleaned after initial filtration using Trimmomatic (v0.36) (Bolger et al., 2014) to remove adapters and low-quality reads. Subsequently, genome assembly was performed using SPAdes (v3.15) (Bankevich et al., 2012), and gap filling was conducted using GapFiller (v1.11) (Bankevich et al., 2012). To ensure high-quality downstream genome assembly and gene annotation, the minimum acceptable fold coverage was predefined as 100×. In this study, the whole-genome sequencing achieved an average depth of approximately 318×, ensuring the reliability of the subsequent genomic characterization. To robustly evaluate the quality of the *de novo* genome assembly, formal quality analyses were conducted. CheckM analysis revealed a high assembly completeness of 96.68% with a remarkably low contamination rate of 0.55%. Furthermore, BUSCO analysis demonstrated a completeness of 100%. These metrics collectively confirm the high reliability of the genome assembly. Gene predictions and annotations were generated using Prokka (Version 1.10) (Seemann, 2014) and the National Center for Biotechnology Information (NCBI) database. The functional annotation of genes primarily relied on protein-coding genes from the Non-Redundant (NR), Gene Ontology (GO) (The Gene Ontology Consortium, 2015), the Kyoto Encyclopedia of Genes and Genomes (KEGG) (Kanehisa and Goto, 2000), Conserved Domain Database (CDD) (Marchler-Bauer et al., 2013), Cluster of Orthologous Groups of proteins (COG) (Tatusov et al., 2000), Carbohydrate-Active Enzymes (CAZy) (Lombard et al., 2014), Protein family (PFAM) (Finn et al., 2016), Pathogen–Host Interactions (PHI) (Urban et al., 2015), and Transporter Classification Database (TCDB). Functional gene annotation was conducted through homology-based searches using DIAMOND (v2.0.8) against multiple specialized databases, including the Comprehensive Antibiotic Resistance Database (CARD) (Mcarthur et al., 2013) for resistance genes and the Virulence Factor Database (VFDB) (Chen et al., 2016) for virulence factors. An E-value threshold of <1e-5 was applied for database searches, with other algorithmic parameters set to default according to the available service workflow. For downstream interpretation, exported CARD and VFDB hits were further curated, and entries with an E-value of <1e-5 and protein identity of >27% were retained. This identity threshold was used to allow detection of distant homologs, given the evolutionary divergence between *Macrococcus* and the well-characterized organisms represented in CARD and VFDB. All retained entries were therefore interpreted as putative homologs identified by homology-based searches, and their precise biological functions require future experimental validation.

### Core-gene analysis

The core genes of *Macrococcus* were analyzed using the Roary pipeline (Page et al., 2015). Core genes were strictly defined as those present in ≥95% of the analyzed strains (95% ≤ strains ≤ 100%), yielding a total of 260 core genes. Concurrently, accessory genes (including shell and cloud genes present in <95% of the strains) were also identified, totaling 12,698 genes. The phylogenetic tree of core-genome sequences was constructed using maximum likelihood with RAxML. The ANI values were calculated using the OrthoANI algorithm (ChunLab). Subsequently, for the high-resolution intra-subspecies analysis, the Panaroo pipeline (Tonkin-Hill et al., 2020) was additionally used among the *M*. *caseolyticus subsp*. *caseolyticus* genomes. This targeted approach was employed to accurately refine the subspecies core genome (yielding 1,717 core genes) and to reconstruct a high-fidelity phylogenetic tree of the clinical isolate and its closest reference genomes.

### Antimicrobial susceptibility test

Antimicrobial susceptibility testing (AST) was conducted using the AutoMic-i600 automatic bacteria identification/drug sensitivity tester (Autobio Diagnostics Co., Ltd, China). The drug sensitivity results were interpreted following the guidelines of the Clinical Laboratory Standards Institute (CLSI standard 2022) (https://clsi.org). Since specific clinical breakpoints for *Macrococcus* species are not currently available, the minimum inhibitory concentrations (MICs) and susceptibility categories (Susceptible, Intermediate, Resistant) were interpreted using the criteria established for *Staphylococcus* species. The tested antimicrobial agents included penicillin, oxacillin, teicoplanin, vancomycin, ciprofloxacin, moxifloxacin, tetracycline, gentamicin, linezolid, clindamycin, and erythromycin. Consistent with the surrogate interpretation strategy mentioned above, *Staphylococcus aureus* ATCC 29213 was selected as the quality control strain, as no genus-specific quality control strains are currently designated for *Macrococcus*.

### Biofilm formation assay

To evaluate the biofilm-forming capacity of the WH712 isolate, a quantitative assay utilizing a 96-well microtiter plate was conducted (Stepanović et al., 2000). Both the clinical isolate WH712 and the positive control strain *Staphylococcus aureus* ATCC 29213 were streaked onto blood agar plates and incubated for 24 h. Individual colonies were suspended in sterile saline, and the turbidity was adjusted to a 0.5 McFarland standard. A 20 µL aliquot of the bacterial suspension was inoculated into 2 mL of Todd-Hewitt Broth (THB) to achieve a 1:100 dilution. Subsequently, 200 µL of the diluted suspension was transferred into a 96-well polystyrene microtiter plate, with uninoculated THB serving as the negative control. Following static incubation at 35 °C for 24 h, the planktonic cultures were discarded, and the wells were gently washed twice with sterile saline to remove non-adherent cells. The adherent biofilms were fixed with 200 μL of methanol per well for 15 min. After methanol removal and air-drying, biofilms were stained with 200 μL of 0.1% crystal violet solution for 15 min at room temperature. Excess dye was removed by gently washing the plates under running water. The crystal violet bound to the biofilms was then solubilized using 200 μL of absolute ethanol for 30 min, and the optical density was measured at 620 nm (OD620 based on the available optical filter of our microplate reader) using a microplate reader (Thermo Fisher Scientific, USA). All assays were performed in six biological replicates.

### Statistical analysis

Statistical analyses were performed using GraphPad Prism software (version 9.5.0, GraphPad Software, USA). Differences in biofilm biomass among groups were assessed using ordinary one-way ANOVA followed by Dunnett’s multiple comparisons test, with the uninoculated THB group used as the control. A p-value of <0.05 was considered statistically significant.

## Results

### Case presentation

A 91-year-old male patient with disturbed consciousness was admitted to our hospital on August 6, 2023. The patient had a 50-year history of hypertension and a 3-year history of cerebral infarction. He was admitted to the local hospital for cerebral infarction treatment a month ago. He developed a fever with a maximum temperature of 38.5°C on the third day of treatment. The laboratory results indicated a white blood cell (WBC) count of 15.47 × 10^9^ cells/L (normal 3.5× 10^9^ - 9.5× 10^9^ cells/L) (with 93% neutrophils). The analysis of inflammatory indicators revealed a procalcitonin (PCT) of 0.33 ng/mL (normal <0.05 ng/mL), a hypersensitive C-reactive protein (CRP) of 15.85 mg/L (normal <6 mg/L), and an Interleukin-6 (IL-6) level of 427.10 pg/mL (normal <7 pg/mL). The troponin and myoglobin levels were also found to be increased. Based on this, the initial diagnosis of the patient was septic shock. Two sets of blood cultures were collected and incubated in the automated monitoring VersaTREK blood culture system (Thermo Fisher Scientific, USA). The blood culture tested positive for bacteria the following day, producing pure cultures of one bacterial morphotype. To exclude other microorganisms, three independent colonies were randomly selected from the culture plates and processed for identification. All three isolates were consistently identified as *M. caseolyticus*. Furthermore, no other aerobic, anaerobic, or fungal microorganisms were detected in either set. Consequently, the patient was diagnosed with monomicrobial septicemia caused by *M. caseolyticus*. The strain was confirmed to be *M. caseolyticus* by MALDI-TOF MS. AST showed that the isolate was susceptible to most tested antimicrobial agents, including penicillin and oxacillin, but exhibited intermediate resistance to clindamycin (**Table 1**). Anti-infective treatment with piperacillin-tazobactam was initiated. However, due to severe underlying comorbidities and a compromised immune system, the patient’s condition rapidly deteriorated. He ultimately succumbed to sepsis and multiple organ failure four days later.

**Table 1:** Antimicrobial susceptibility of *M. caseolyticus* subsp. *caseolyticus*.

Antimicrobial agent	MIC (μg/ml)	Interpretation (SIR)
Penicillin	<=0.12	S
Oxacillin	0.5	S
Teicoplanin	<=1	S
Vancomycin	<=0.5	S
Ciprofloxacin	0.25	S
Moxifloxacin	0.25	S
Tetracycline	<=1	S
Gentamicin	<=2	S
Linezolid	<=1	S
Clindamycin	1	I
Erythromycin	<=0.25	S

MIC, the minimum inhibitory concentration; SIR, (S) susceptible, (I) intermediate, (R) resistant.

### Microbiological characteristics of *M. caseolyticus*

**Figure 1:**
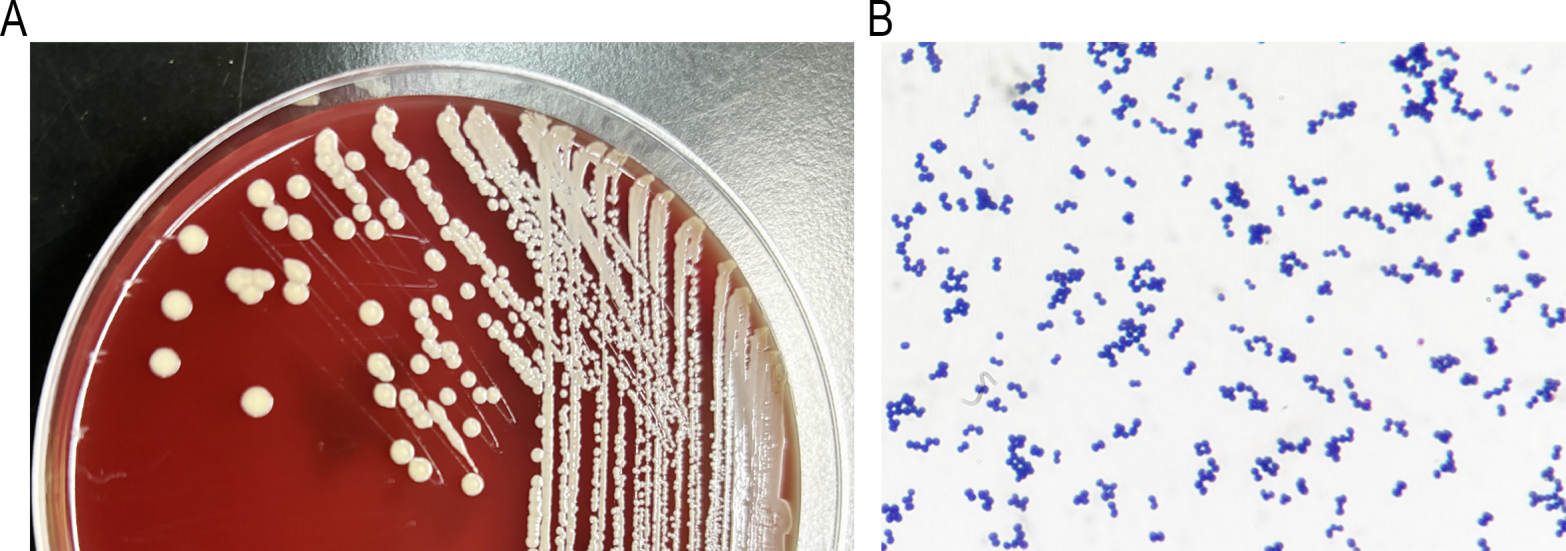
The growth conditions of *M. caseolyticus* subsp. *caseolyticus* strain WH712. **(A)** Growth of *M. caseolyticus* subsp. *caseolyticus* on blood agar media. **(B)** Gram staining reveals Gram-positive cocci arranged in clusters (1000× magnification).

Following overnight incubation, multiple colonies with a golden yellow, round appearance were observed on the Columbia blood agar plate (**Figure 1A**). The microscopic examination revealed the presence of Gram-positive cocci (**Figure 1B**). These findings indicated that the patient had a bloodstream infection. Individual colonies were identified as *M. caseolyticus* using MALDI-TOF MS with a confidence level of 99.9%. A spectrum of protein molecular mass was obtained for this strain. The peaks in the mass spectra represented the relative intensity of ions. The molecular structure and composition of compounds were determined by analyzing the position and size of the peaks. The pattern-matching procedure was employed to compare the mass peaks in the experimental spectra with those in the reference spectra in the manufacturer’s database (bioMérieux, France).

### Core-gene sequences analysis

**Figure 2:**
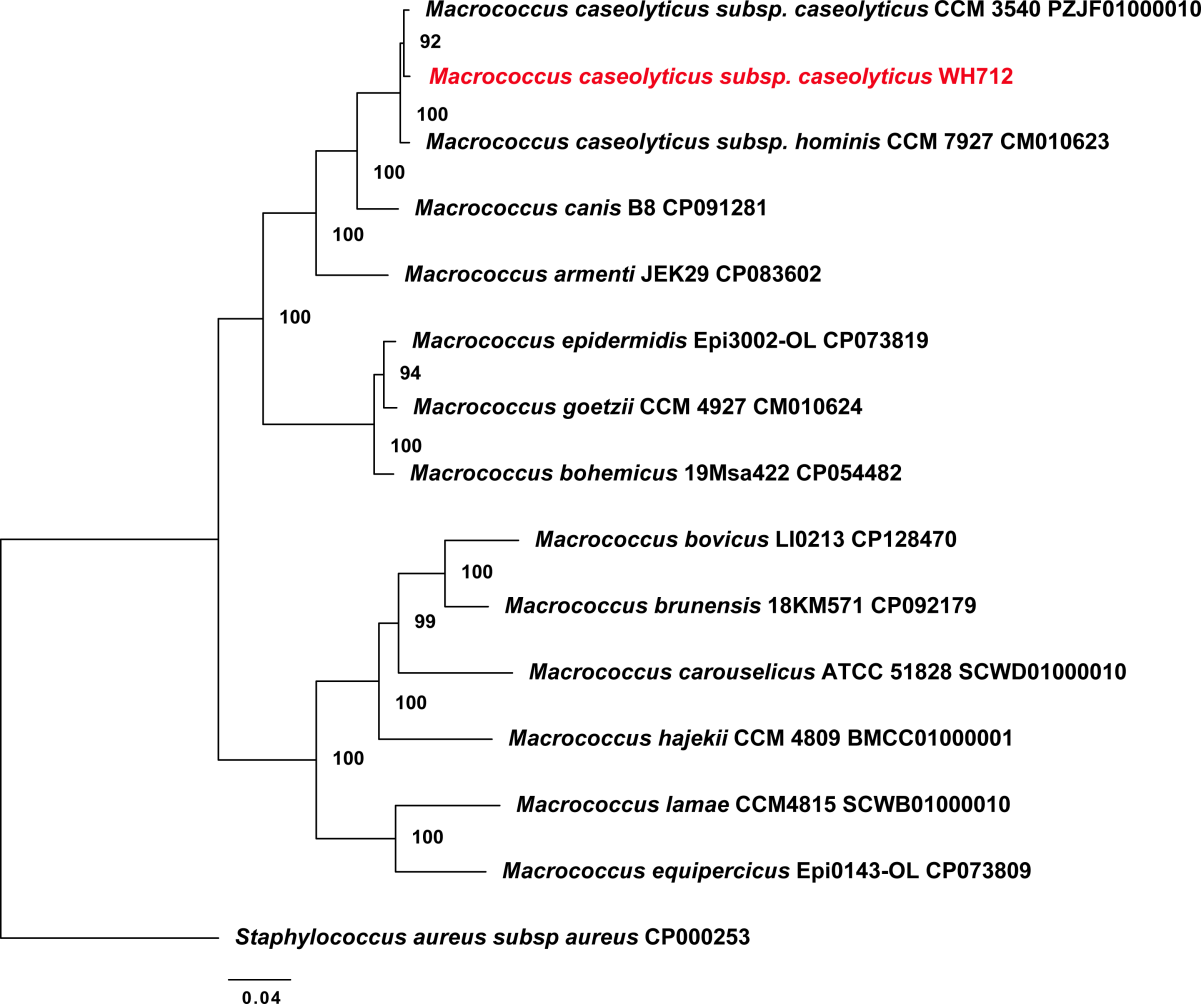
Core-genome-based phylogenetic tree of Macrococcus species. The position of the clinical isolate *M. caseolyticus* subsp. *caseolyticus* WH712 is highlighted in red.

**Figure 3:**
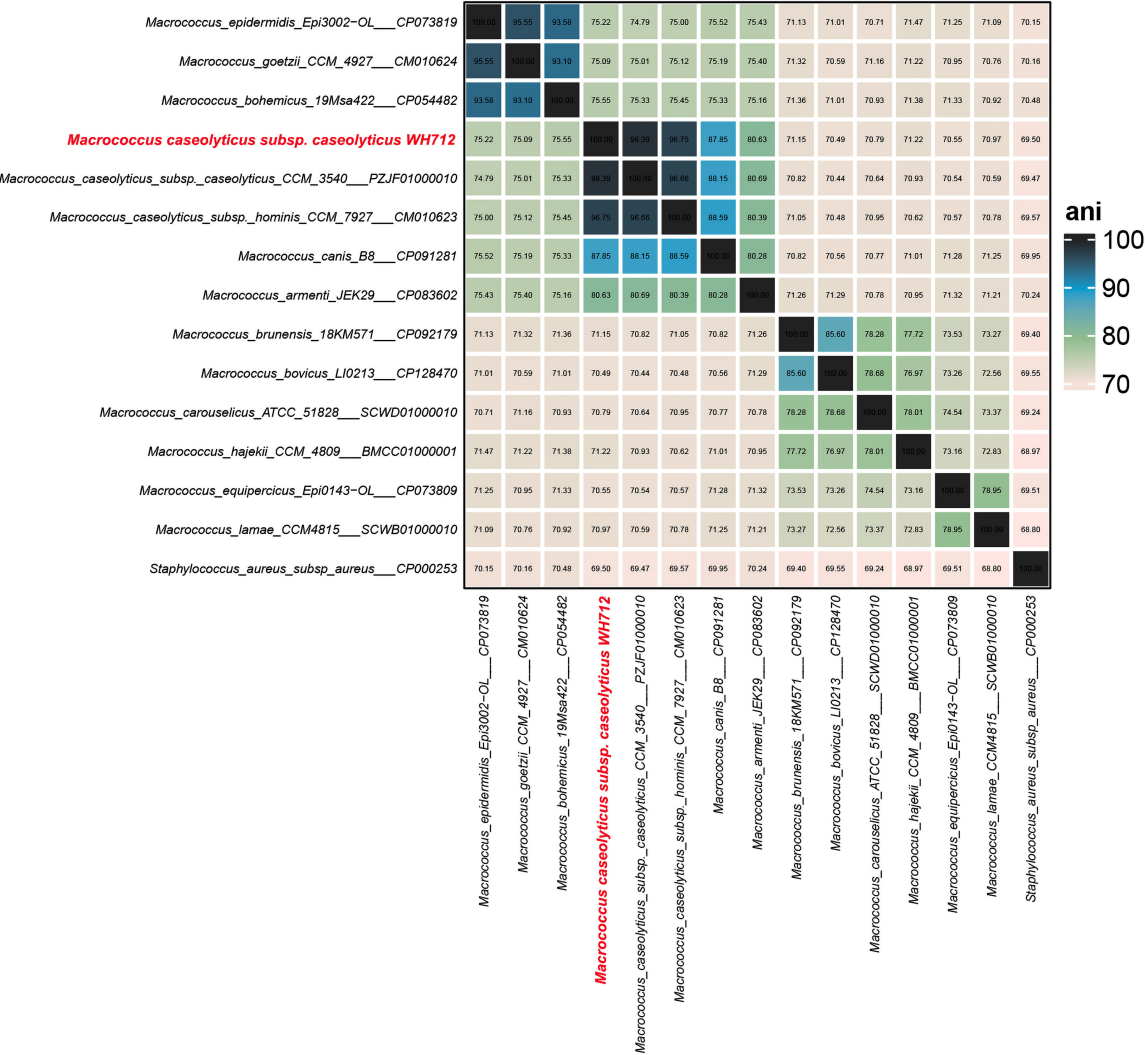
Average nucleotide identity (ANI) heatmap illustrating the taxonomic relationships among *Macrococcus* species. The clinical isolate WH712 (highlighted in red) shares the highest ANI with the *M. caseolyticus* subspecies.

To gain deeper genomic insights and definitively confirm the taxonomic status of strain WH712, pangenome and core-genome analyses were performed. The pangenome analysis using Roary identified a pan-genome of 12,958 genes, comprising 260 orthologous core genes (present in ≥95% of strains) and 12,698 accessory genes (including 3,500 shell genes and 9,198 cloud genes). Based on the concatenated alignment of single-copy core genes, a maximum-likelihood phylogenetic tree was constructed. This core-genome phylogeny robustly clustered strain WH712 with the *M. caseolyticus* subsp. *caseolyticus* type strain CCM 3540 (**Figure 2**). This whole-genome-level classification was further corroborated by the Average Nucleotide Identity (ANI) analysis. Strain WH712 exhibited an ANI value of 98.39% with *M. caseolyticus* subsp. *caseolyticus*, which is well above the recognized species demarcation threshold of 95%, whereas it shared only 96.75% ANI with *M. caseolyticus* subsp. *hominis* (**Figure 3**).

Building upon this taxonomic confirmation, we further applied the rigorous Panaroo pipeline to perform a targeted intra-subspecies pangenome analysis among the eight most closely related *M*. *caseolyticus* subsp. *caseolyticus* strains. This micro-evolutionary analysis identified a robust set of 1,717 core genes within the subspecies. The resulting high-fidelity phylogenetic tree based on this Panaroo output delineated the evolutionary position of our clinical isolate among its closest relatives (**Supplementary Figure 3**).

### WGS of *M. caseolyticus* subsp. *caseolyticus*

**Figure 4:**
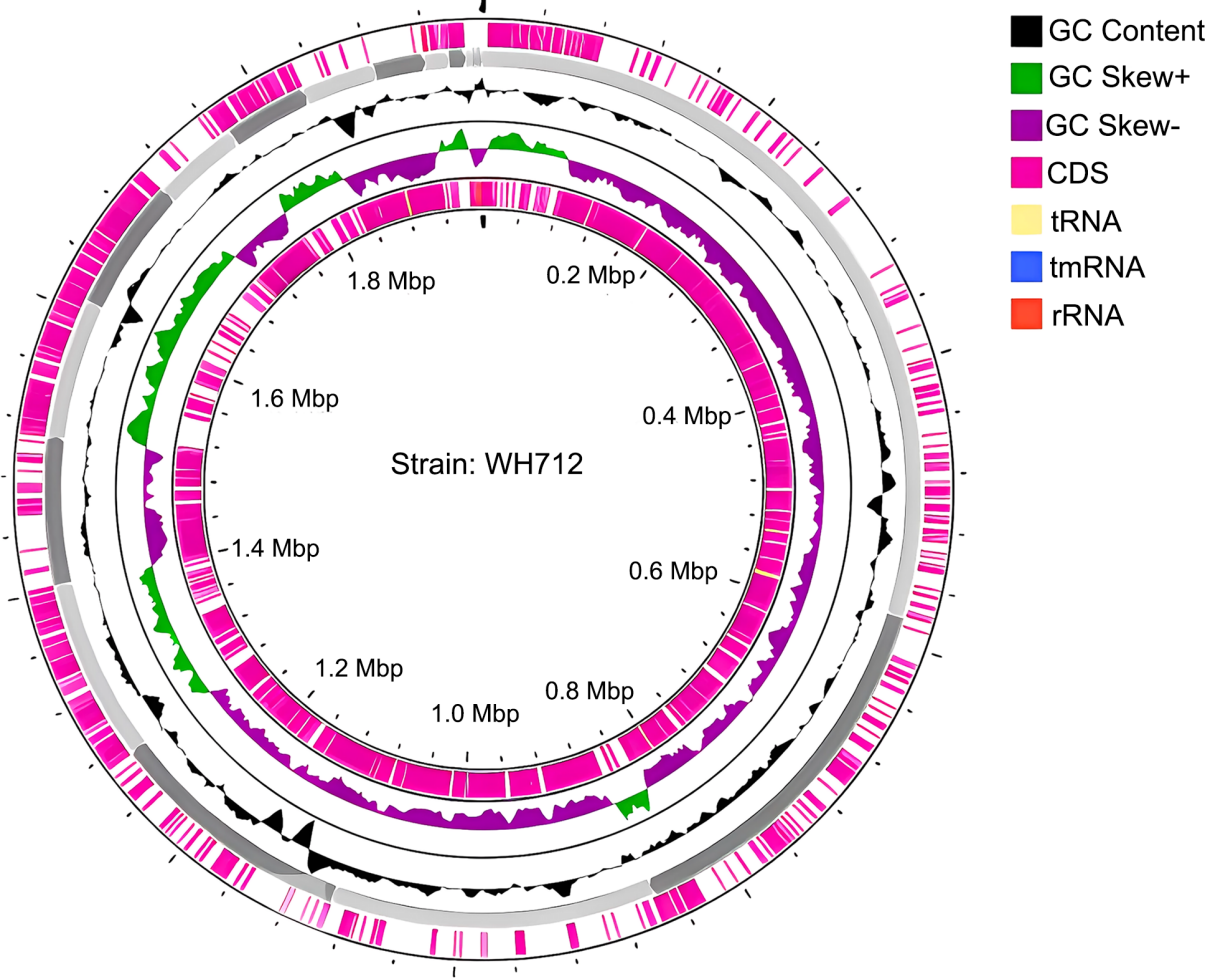
The map showing the assembled genome sequence of *M. caseolyticus* subsp. *caseolyticus* strain WH712. From outside to inner side rings: the coding sequences (CDS) in pink, RNA genes (tRNAs in yellow, tmRNA in blue, and rRNAs in red), GC content in black, and GC-skew graph in green and purple.

Fatal *M. caseolyticus* bloodstream infections in humans have not been previously reported. To enhance our understanding of the pathogens involved in the current cases, the strain was subjected to whole-genome sequencing. The draft genome sequence of *M. caseolyticus* subsp. *caseolyticus* WH712 was found to be 1,962,099 bp in size and assembled into 19 contigs. The N50 value of the assembly was determined to be 234,236 bp. The GC content of the assembled genome was 36.93% (**Figure 4**), which aligned with previous reports. The NCBI PGAP tool was used for automatic annotation, resulting in the identification of 2006 protein-coding genes. The OrthoANIu algorithm was employed to confirm the taxonomic classification of WH712 as *M. caseolyticus*, revealing a high ANI value of 98.39% between WH712 and the *M. caseolyticus* subsp. *caseolyticus* strain CCM 3540. Additionally, the DNA–DNA hybridization (DDH) value between strain WH712 and its related type strain was analyzed using Genome-to-Genome Distance Calculator (GGDC) 3.0, yielding an estimated value of 98.80%. Protein-coding genes were annotated using the NR, COG, PFAM, CDD, KEGG, GO, PHI, TCDB, and Cazy databases. The annotations resulted in 2002 (99.90%) genes from NR, 1558 (77.74%) genes from COG, 1406 (70.16%) genes from PFAM, 939 (46.86%) genes from CDD, 708 (35.33%) genes from KEGG, 509 (25.40%) genes from GO, 395 (19.71%) genes from PHI, 245 (12.23%) genes from TCDB, and 23 (1.15%) genes from CAZy.

### Prediction of pathogenicity and antibiotic resistance

**Figure 5:**
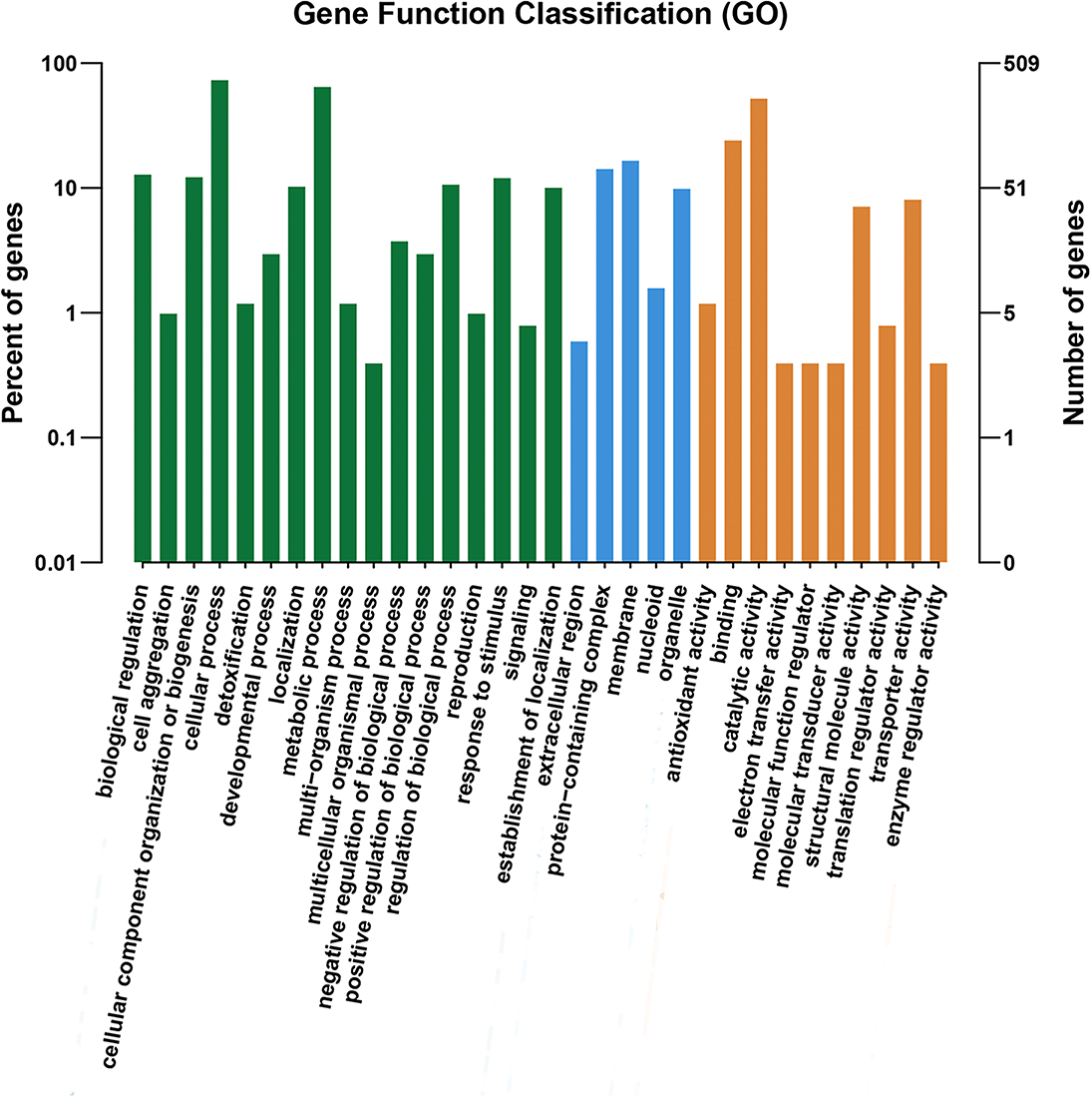
Functional annotation of proteins encoded by *M. caseolyticus* subsp. *caseolyticus* genes using the GO database elucidates the relationship between genes and gene products. The left axis represents the percentage of genes, while the right axis displays the gene numbers.

Homology-based searches against the CARD database identified multiple putative antibiotic resistance-associated homologs in strain WH712, including hits related to antibiotic efflux, target alteration, target protection, and target inactivation (**Supplementary Table 1**). Similarly, searches against the VFDB database identified multiple putativevirulence-associated homologs (**Supplementary Table 2**). Among these, homologs relevant to capsule-associated functions and stress responses were detected, and additional putative homologs potentially related to adhesion, biofilm formation, and persistence were also identified. The WH712 genome also contained putative homologs of *hlyB*, *cpsA*, *cpsJ*, *cap8D*, and *cap8P*. Because these annotations were inferred from homology-based searches, their precise biological roles in *M*. *caseolyticus* remain to be experimentally validated. GO annotation of the predicted *M. caseolyticus* subsp. *caseolyticus* WH712 genes resulted in the identification of 1121 genes enriched in terms related to biological processes, 217 genes in the category of molecular function, and 481 genes in the category of cellular component (**Figure 5**). Therefore, many of these metabolic genes might be involved in signal transduction. The KEGG functional classification indicated that the predicted *M. caseolyticus* subsp*. caseolyticus* WH712 genes were predominantly involved in metabolism (965 genes), genetic information processing (195 genes), environmental information processing (155 genes), cellular processes (46 genes), and organismal systems (28 genes). The enrichment of functions related to metabolism and genetic information processing suggests that these genes may play a role in facilitating efficient metabolism and information exchange (**Supplementary Figure 1**). The sequences of *M. caseolyticus* subsp. *caseolyticus* WH712 were aligned to obtain annotated results from the PHI database. A total of 433 genes related to human diseases were identified, including skin infections, food poisoning, respiratory diseases (90 genes), and nosocomial infections (84 genes) (**Supplementary Figure 2**). These results suggested that *M. caseolyticus* subsp. *caseolyticus* might have the potential to be a pathogen in humans. To further evaluate its pathogenic potential, the whole genome of strain WH712 was analyzed using the PathogenFinder 2.0 web server. The computational model yielded a mean prediction score of 0.3469, classifying the isolate as having ‘Human Non Pathogenic’ capacity. Interestingly, this *in silico* prediction contrasts starkly with the fatal clinical outcome observed in our patient. This discrepancy likely stems from the fundamental nature of the predictive model, which relies heavily on training datasets of classical, well-documented human pathogens. As *M. caseolyticus* is traditionally an animal commensal, its unique, emerging virulence determinants are likely underrepresented in current human pathogen databases. Furthermore, the model predicts primary pathogenicity, whereas the fatal infection in this study was opportunistic, critically facilitated by the 91-year-old patient’s severely immunocompromised status. This finding suggests that current *in silico* pathogenicity prediction tools may have limited performance for rare or emerging opportunistic organisms such as *M. caseolyticus*, particularly when host susceptibility plays a major role.

### Phenotypic validation of biofilm production

**Figure 6:**
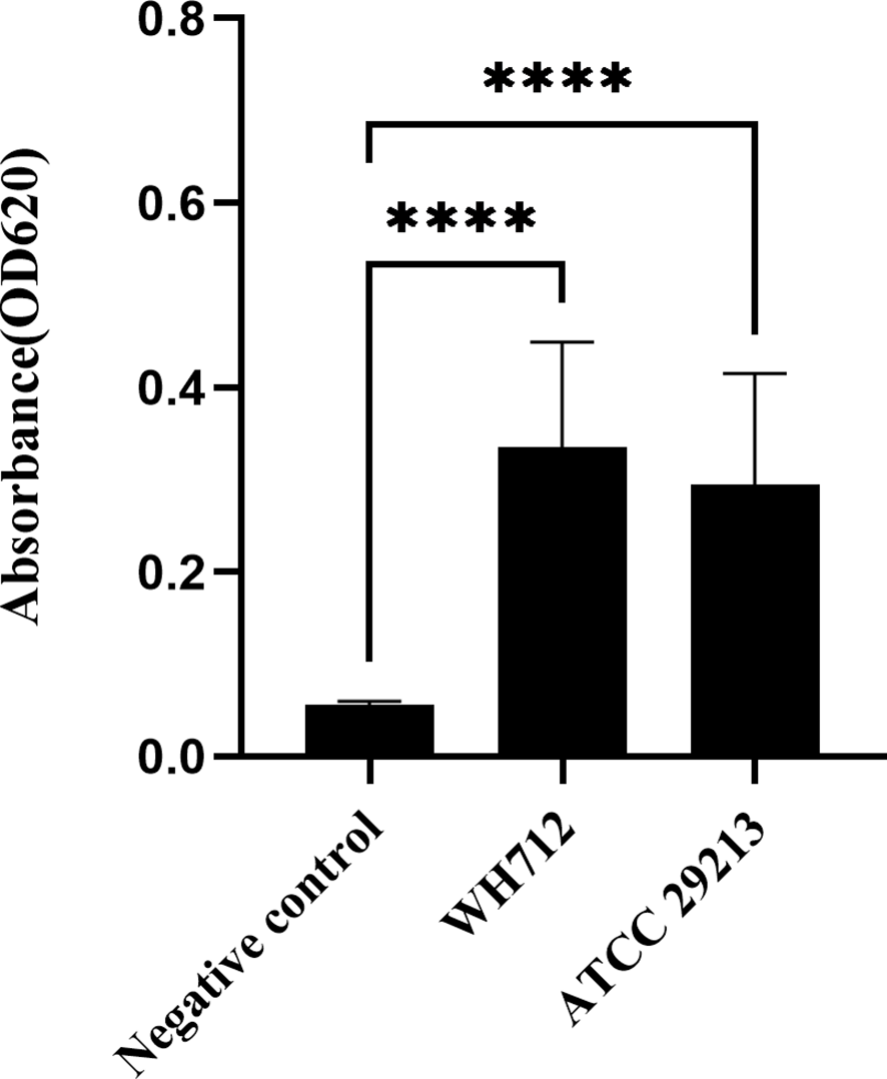
Phenotypic validation of biofilm formation in *M. caseolyticus* subsp. *caseolyticus* WH712 was conducted. The X-axis represents the experimental groups, and the Y-axis shows biofilm biomass, quantified by absorbance at OD620 after staining with 0.1% crystal violet. Statistical significance was determined using ordinary one-way ANOVA followed by Dunnett’s multiple comparisons test, with the uninoculated THB group used as the control. **** P < 0.0001.

Consistent with the genomic identification of multiple biofilm-associated genes, the phenotypic microtiter plate assay confirmed that *M*. *caseolyticus* subsp. *caseolyticus* WH712 is a strong biofilm producer. Quantitative analysis employing 0.1% crystal violet staining demonstrated that the mean optical density at 620 nm (OD620) of the WH712 strain was significantly elevated compared to the negative control (uninoculated THB) (P < 0.0001). According to established classification criteria, WH712 was classified as a strong biofilm producer because its OD620 value exceeded four times the cut-off OD value (ODc), which was calculated from the negative control. Notably, its biofilm-forming ability was robust and comparable to that of the well-documented strong biofilm producer *Staphylococcus aureus* ATCC 29213, which served as a positive control (**Figure 6**).

## Discussion

As a member of the family *Staphylococcaceae*, the genus *Macrococcus* is closely related to *Staphylococcus* but has traditionally been regarded as having limited pathogenicity in humans. These bacteria are primarily recognized as commensals of animals and as organisms associated with food products, although they may occasionally act as opportunistic pathogens in animals (Gómez-Sanz et al., 2015; Hansen et al., 2015). The clinical relevance of this genus in highly susceptible hosts may therefore be underrecognized. A recent study reported that *M. caseolyticus* caused highly fatal infections in commercial broiler chickens, characterized by severe caseous exudation in cranial cavities, inflammatory infiltration, hemorrhages, and multifocal necrosis across various organs (Li et al., 2018). Furthermore, the recovery of four *M. caseolyticus* subsp. *hominis* strains from human clinical specimens suggests that members of this species may occasionally be encountered in human clinical settings (Mašlaňová et al., 2018). Similar clinical scenarios have been described for other traditionally low-virulence or commensal bacteria. Multidrug-resistant *Corynebacterium striatum* has been reported to cause fatal sepsis in an elderly patient with myelodysplastic syndrome and corticosteroid exposure (Chatzopoulou et al., 2016). In addition, *Micrococcus luteus*, a skin commensal often regarded as a contaminant, has been reported to cause native valve infective endocarditis in a patient with non-Hodgkin’s lymphoma (Ianniello et al., 2019). These reports support the concept that severe infections caused by uncommon organisms often reflect the combined effects of host susceptibility, underlying disease, invasive procedures, and microbial persistence rather than high intrinsic bacterial virulence alone.

Within this framework, the primary objective of this report was to describe the clinical course, empirical antibiotic therapy, and genomic/phenotypic features of a fatal bloodstream infection associated with *M. caseolyticus* subsp. *caseolyticus* in China. By presenting this case, we aim to provide clinically relevant observations for interpreting rare opportunistic infections in highly susceptible patients.

In the present case, empirical therapy with piperacillin-tazobactam was administered. Although piperacillin-tazobactam has broad-spectrum activity, piperacillin was originally developed with an important role in Gram-negative coverage, including activity against *Pseudomonas aeruginosa*, and its clinical efficacy against *Macrococcus* species has not been established. Although the WH712 isolate demonstrated *in vitro* susceptibility to penicillin and oxacillin under surrogate *staphylococcal* interpretation, suggesting the absence of a methicillin-resistant phenotype, the clinical condition rapidly deteriorated into septic shock and multiple organ failure. This discrepancy between the isolate’s susceptible profile and the fatal outcome warrants careful interpretation. The patient’s advanced age, severe underlying comorbidities, and impaired host defenses likely contributed substantially to the unfavorable outcome. Therefore, therapeutic failure and mortality were likely driven primarily by the patient’s advanced age, severe comorbidities, impaired host defenses, and progression to septic shock, rather than by the intrinsic virulence of the strain alone. Overall, the fragile clinical status of the patient was likely a major determinant of the fatal outcome, highlighting the potential clinical impact of opportunistic organisms in highly vulnerable populations.

Given that *M. caseolyticus* subsp. *caseolyticus* is rarely encountered in human bloodstream infections, we conducted a comprehensive screening for homologs of known virulence- and resistance-associated factors to explore potential bacterial features that might have contributed to colonization or persistence in this vulnerable host.

Whole-genome analysis provided additional clues to the biological features of WH712. Several putative homologs associated in other bacteria with adhesion, cell-surface anchoring, and biofilm-related regulation, including *ami*, *srtE*, *pfbA*, and *bopD*, were identified by homology-based searches. The detected *ami* homolog may be relevant to initial bacterial attachment (Heilmann et al., 1997), whereas the *srtE* homolog may be associated with cell-surface protein anchoring (Tsubakishita et al., 2010). A relatively higher-identity *bopD* homolog was also identified; in *Enterococcus faecalis*, this gene has been associated with biofilm-related regulation (Hufnagel et al., 2004). In addition, the detected *pfbA* homolog may suggest a potential role in host interaction and persistence (Yamaguchi et al., 2008). Overall, these findings suggest that WH712 may harbor genomic features relevant to adhesion and biofilm-associated persistence, although further functional validation is required. Genomic analysis also identified homologs of *arlR*, *arlS*, and *mgrA* in the WH712 genome, suggesting the presence of an *ArlRS*-*MgrA*-like regulatory system. In related members of the *Staphylococcaceae*, this regulatory system has been implicated in multidrug efflux, biofilm development, and host colonization (Fournier et al., 2000; Truong-Bolduc et al., 2005; Burgui et al., 2018; Crosby et al., 2020). Whether it plays a similar role in *M. caseolyticus*, however, remains to be determined.

Beyond virulence- and colonization-associated features, the genomic profile of WH712 may also be relevant to its antimicrobial phenotype. The intermediate resistance to clindamycin observed in this isolate may be associated with the presence of the *vgaB* homolog, which has been linked to ABC-F-mediated protection against lincosamides in other bacteria (Tessé et al., 2013). In addition, the genomic findings provided a rationale for further phenotypic assessment of biofilm formation.

This interpretation was further supported by the *in vitro* phenotypic assay, which showed that strain WH712 had a strong biofilm-forming capacity (**Figure 6**). Biofilm formation may facilitate bacterial persistence by limiting antimicrobial penetration and reducing susceptibility to host immune clearance. In this case, the biofilm-forming phenotype might have contributed to colonization or persistence of the strain *in vivo*. However, the discrepancy between the broadly susceptible *in vitro* AST profile and the fatal clinical outcome should be interpreted primarily through the lens of host vulnerability. These findings suggest that bacterial traits such as biofilm formation may enable opportunistic persistence, but the clinical trajectory of such rare infections should be interpreted in the broader context of host immune status, comorbidities, and empirical treatment choices.

In conclusion, we report a rare fatal bloodstream infection associated with *M. caseolyticus* subsp. *caseolyticus* in a highly vulnerable elderly patient. Genomic screening and phenotypic assays identified bacterial features, including strong biofilm-forming capacity, that may have facilitated colonization or persistence. However, the fatal clinical outcome should be interpreted primarily in the context of the patient’s advanced age, severe comorbidities, septic shock, and impaired host defenses, rather than as evidence of high intrinsic bacterial virulence. This case highlights that organisms with typically low pathogenic potential may cause clinically significant infections in highly susceptible hosts. It also underscores the need to interpret conventional *in vitro* AST results together with host factors, empirical treatment choices, and genomic/phenotypic characteristics when evaluating severe infections caused by uncommon organisms.

## Data Availability

The datasets presented in this study can be found in online repositories. The names of the repository/repositories and accession number(s) can be found in the article/**Supplementary Material**.
