# Supplementary material for: First fatal human bloodstream infection caused by Macrococcus caseolyticus subsp. caseolyticus in China: genomic insights into virulence and antimicrobial resistance
Source: Front Cell Infect Microbiol. 2026 Jun 1;16:1825695. doi: 10.3389/fcimb.2026.1825695 (PMC13265326; doi:10.3389/fcimb.2026.1825695)
Supplement: Supplementary file 1 [file Table1.docx]

**First fatal human bloodstream infection caused by *Macrococcus* *caseolyticus* subsp. *caseolyticus* in China: Genomic insights into virulence and antimicrobial resistance**


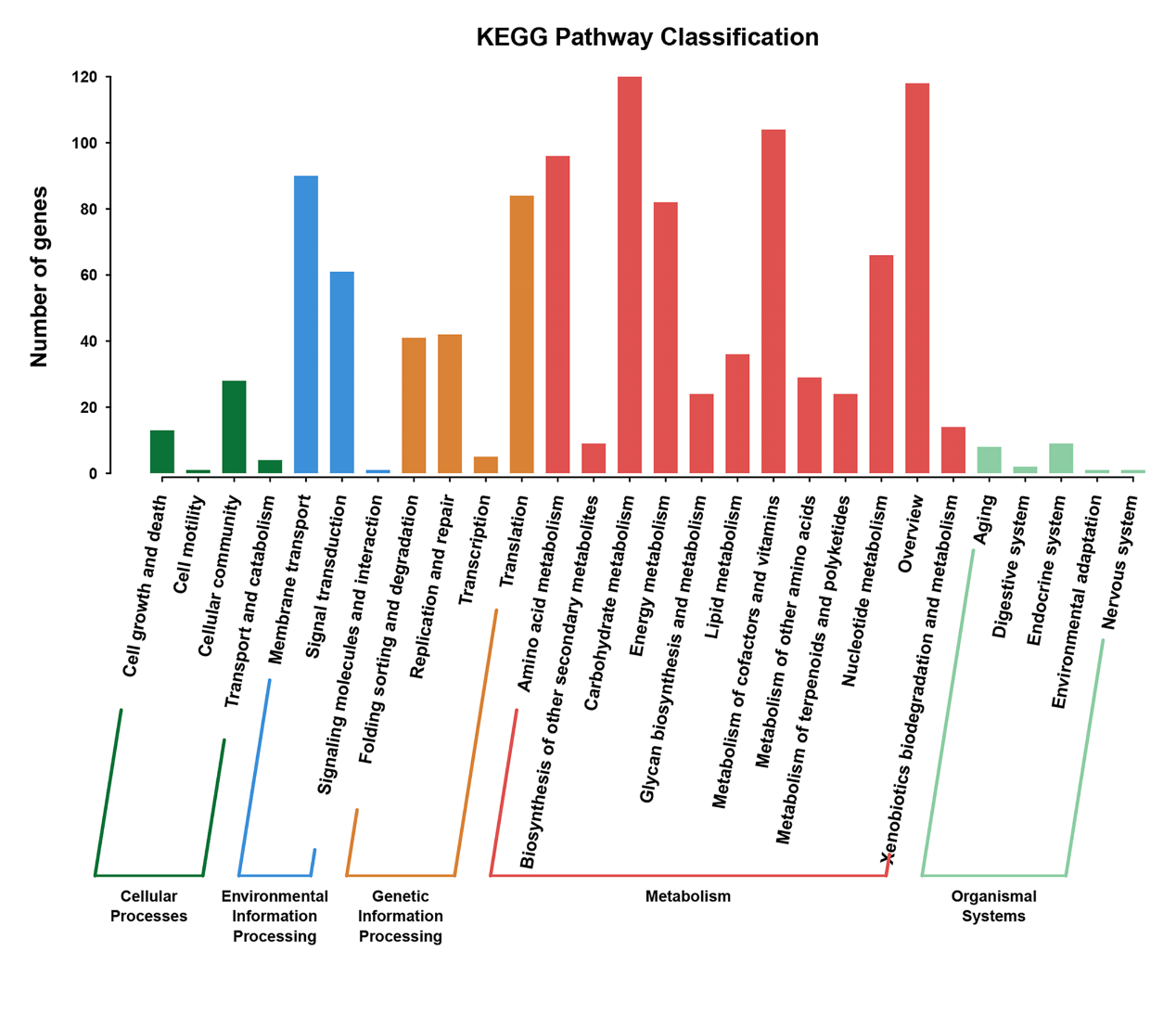


**Figure S1.** Functional annotation of *M*. *caseolyticus* subsp. *caseolyticus* gene-encoding proteins using KEGG reveal the predicted genes associated with human diseases and metabolic pathways. The vertical axis displays the number of genes annotated to each pathway, while the horizontal axis represents the names of metabolic pathways.


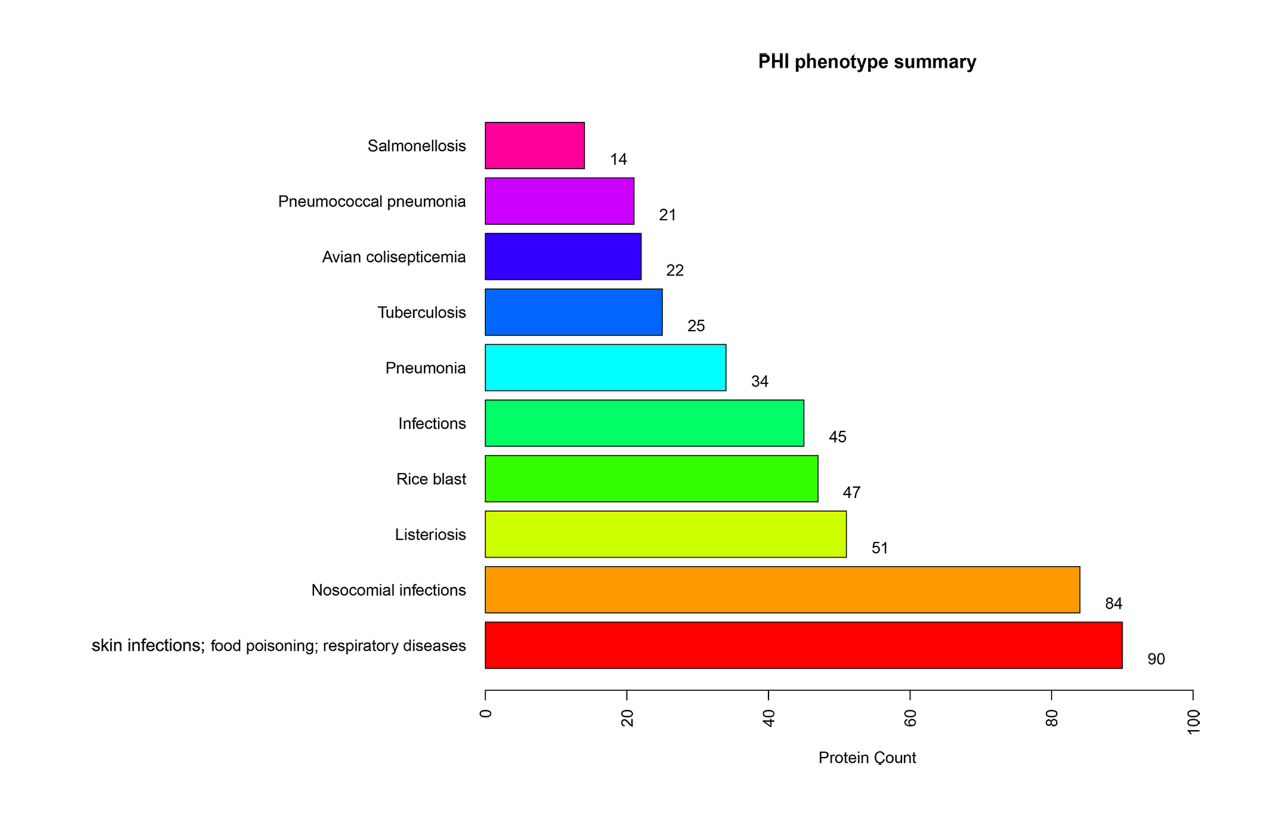


**Figure S2.** Functional annotation of *M. caseolyticus* subsp. *caseolyticus* gene-encoding proteins was performed using the PHI database to predict pathogen-host interactions.


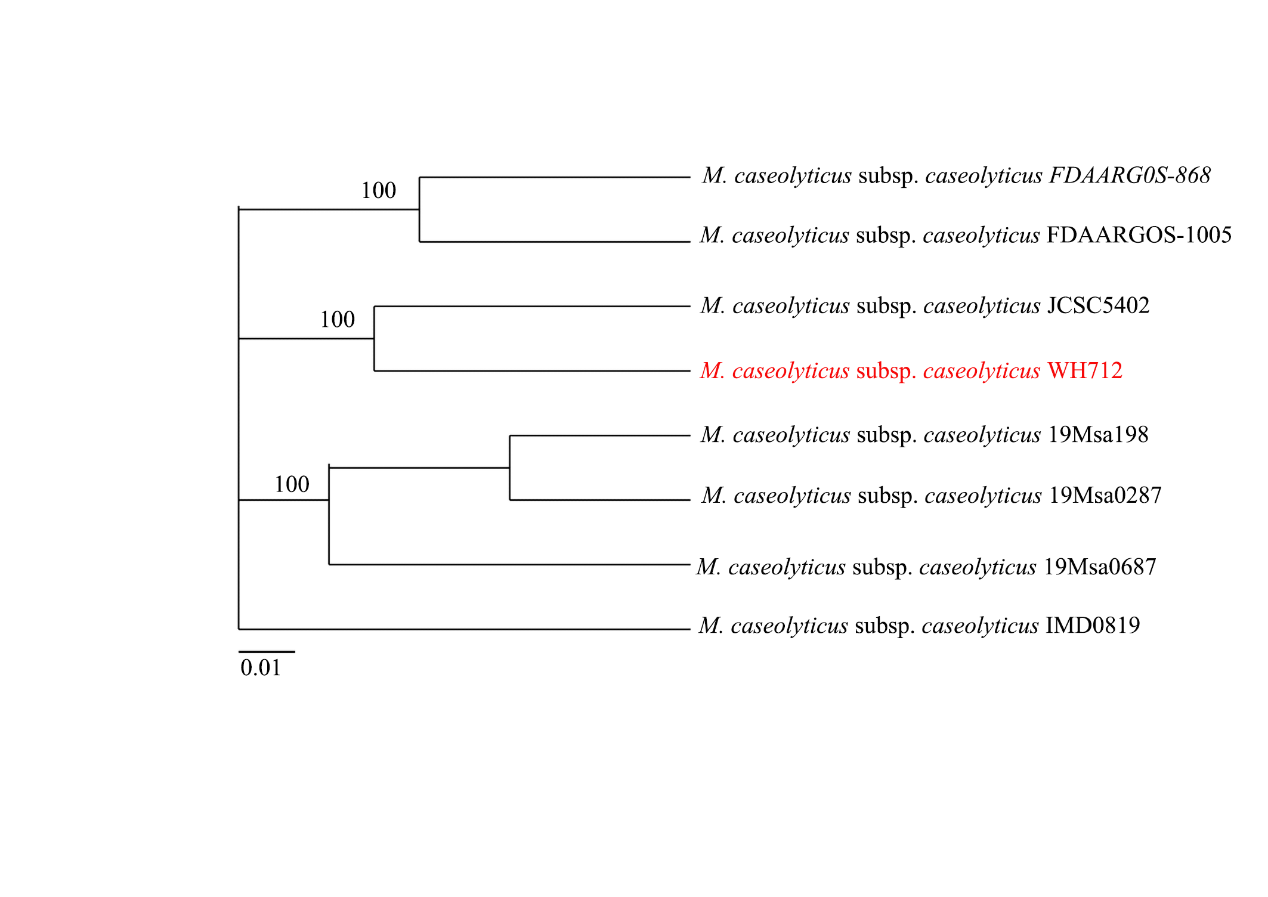


**Figure S3.** Intra-subspecies core-genome phylogenetic tree of Macrococcus caseolyticus subsp. caseolyticus strains generated using Panaroo.

The tree was constructed from the alignment of 1,717 core genes identified among eight M. caseolyticus subsp. caseolyticus genomes. The clinical isolate WH712 is highlighted in red.
