# Supplementary material for: First fatal human bloodstream infection caused by Macrococcus caseolyticus subsp. caseolyticus in China: genomic insights into virulence and antimicrobial resistance
Source: Front Cell Infect Microbiol. 2026 Jun 1;16:1825695. doi: 10.3389/fcimb.2026.1825695 (PMC13265326; doi:10.3389/fcimb.2026.1825695)
Supplement: Supplementary file 2 [file Table2.docx]

**Table S1.** Putative antibiotic resistance gene homologs detected in *M*. *caseolyticus* subsp. *caseolyticus* using the CARD database.

| **CARD ID** | **Gene Name** | **Identity (%)** | **E-value** | **Resistance mechanism** |
| --- | --- | --- | --- | --- |
| ARO:3000815  ARO:3004480  ARO:3003063  ARO:3000839  ARO:3002569  ARO:3004036  ARO:3002986  ARO:3007012  ARO:3007013  ARO:3007191  ARO:3002922  ARO:3002922  ARO:3000535  ARO:3004361  ARO:3002926  ARO:3002987  ARO:3004572  ARO:3002987  ARO:3002956  ARO:3003069  ARO:3003746  ARO:3003785  ARO:3003105  ARO:3004047  ARO:3002926  ARO:3002926  ARO:3003324  ARO:3002522  ARO:3005091  ARO:3007050  ARO:3002944  ARO:3000010  ARO:3003948  ARO:3000828  ARO:3000118  ARO:3000815  ARO:3000535  ARO:3003948  ARO:3002522  ARO:3000553  ARO:3007188  ARO:3002522  ARO:3002974  ARO:3002926  ARO:3002522  ARO:3003746  ARO:3003950  ARO:3000521  ARO:3003006  ARO:3004035  ARO:3004035  ARO:3003060  ARO:3002987  ARO:3002972  ARO:3006873  ARO:3004442  ARO:3003986  ARO:3003578  ARO:3003969  ARO:3000025  ARO:3000024  ARO:3003950  ARO:3000838  ARO:3002702  ARO:3004033  ARO:3003948 | mgrA  Bado_rpoB_RIF  ykkC  arlS  AAC(6')-Iy  tetB(60)  bacA  sepA  sdrM  vanR_in_vanP_cl  vanR_in_vanC_cl  vanR_in_vanC_cl  macB  sul4  vanR_in_vanG_cl  bcrA  Saur_LmrS  bcrA  vanY_in_vanB_cl  vanXY_in_vanG  optrA  Ctra_murA_FOF  dfrA3  kdpD  vanR_in_vanG_cl  vanR_in_vanG_cl  Bsub_mprF  novA  RanA  mreA  vanH_in_vanD_cl  vanA  efrA  baeR  vgaB  mgrA  macB  efrA  novA  adeR  vanH_in_vanP_cl  novA  vanTr_in_vanL  vanR_in_vanG_cl  novA  optrA  msbA  Saur_mupA_MUP  blt  tetA(60)  tetA(60)  tsnR  bcrA  vanT_in_vanG_cl  EAM-1  tet(W/N/W)  TaeA  PmrF  lmrP  patB  patA  msbA  arlR  cmrA  tetB(46)  efrA | 64.5  53.3  51.4  49  46.5  46  46  45  43.4  41.5  40.7  40.6  39  38.9  37.7  37.1  36.4  36.1  36.1  36  35.9  35.6  35.6  35.1  34.5  34.5  34.3  34.3  34.3  34  33.6  33.2  33.2  33  32.6  32.3  32  32  31.5  31.4  31.3  31.1  30.9  30.9  30.9  30.8  29.8  29.7  29.5  29.3  29.3  29  28.9  28.7  28.4  28.1  28.1  27.7  27.5  27.5  27.5  27.4  27.4  27.3  27.2  27.2 | 1.81E-59  0  2.05E-34  5.04E-137  6.67E-29  7.09E-164  2.81E-70  1.83E-45  2.35E-111  1.32E-56  7.08E-54  3.64E-56  2.72E-55  6.08E-59  2.46E-43  3.89E-64  8.40E-91  8.22E-50  1.72E-25  1.27E-25  1.60E-120  2.77E-67  5.47E-31  2.56E-160  4.23E-40  4.31E-39  3.26E-141  1.65E-95  1.67E-39  9.26E-55  3.39E-30  3.91E-52  5.79E-27  3.59E-09  8.15E-82  2.79E-18  2.62E-21  1.68E-24  6.41E-88  8.42E-35  1.25E-48  1.88E-89  2.89E-49  1.92E-37  6.44E-17  1.34E-80  1.25E-24  2.40E-119  2.81E-44  7.45E-14  2.97E-17  2.91E-15  1.89E-36  9.11E-25  1.78E-08  4.55E-81  7.31E-16  5.66E-38  2.04E-39  1.78E-17  1.24E-17  7.00E-21  2.62E-08  1.04E-24  2.09E-17  2.73E-15 | antibiotic efflux  antibiotic target alteration  antibiotic efflux  antibiotic efflux  antibiotic inactivation  antibiotic efflux  antibiotic target alteration  antibiotic efflux  antibiotic efflux  antibiotic target alteration  antibiotic target alteration  antibiotic target alteration  antibiotic efflux  antibiotic target replacement  antibiotic target alteration  antibiotic efflux  antibiotic efflux  antibiotic efflux  antibiotic target alteration  antibiotic target alteration  antibiotic target protection  antibiotic target alteration  antibiotic target replacement  antibiotic efflux  antibiotic target alteration  antibiotic target alteration  antibiotic target alteration  antibiotic efflux  antibiotic efflux  antibiotic efflux  antibiotic target alteration  antibiotic target alteration  antibiotic efflux  antibiotic efflux  antibiotic target protection  antibiotic efflux  antibiotic efflux  antibiotic efflux  antibiotic efflux  antibiotic efflux  antibiotic target alteration  antibiotic efflux  antibiotic target alteration  antibiotic target alteration  antibiotic efflux  antibiotic target protection  antibiotic efflux  antibiotic target alteration  antibiotic efflux  antibiotic efflux  antibiotic efflux  antibiotic target alteration  antibiotic efflux  antibiotic target alteration  antibiotic inactivation  antibiotic target protection  antibiotic efflux  antibiotic target alteration  antibiotic efflux  antibiotic efflux  antibiotic efflux  antibiotic efflux  antibiotic efflux  antibiotic efflux  antibiotic efflux  antibiotic efflux |

Hits were retained for downstream interpretation using protein identity >27% and E-value <1e-5.
