# Supplementary material for: First fatal human bloodstream infection caused by Macrococcus caseolyticus subsp. caseolyticus in China: genomic insights into virulence and antimicrobial resistance
Source: Front Cell Infect Microbiol. 2026 Jun 1;16:1825695. doi: 10.3389/fcimb.2026.1825695 (PMC13265326; doi:10.3389/fcimb.2026.1825695)
Supplement: Supplementary file 3 [file Table3.docx]

**Table S2.** Putative virulence-associated gene homologs detected in *M. caseolyticus* subsp. *caseolyticus* using the VFDB database.

| **VFDB ID** | **Gene Name** | **Identity (%)** | **E-value** | **Function** |
| --- | --- | --- | --- | --- |
| VFG000077  VFG011430  VFG037028  VFG002197  VFG001300  VFG001312  VFG001855  VFG002190  VFG037100  VFG000964  VFG000964  VFG002181  VFG002158  VFG000964  VFG001298  VFG000964  VFG037386  VFG001867  VFG002181  VFG001359  VFG000320  VFG002189  VFG011399  VFG018241  VFG001297  VFG002160  VFG001386  VFG005767  VFG001299  VFG002161  VFG037028  VFG001826  VFG000077  VFG000670  VFG037100  VFG032992  VFG002184  VFG001386  VFG037100  VFG016338  VFG014950  VFG001386  VFG013365  VFG000307  VFG001386  VFG000344  VFG002480  VFG001206  VFG001859  VFG041304  VFG000344  VFG000344  VFG013682  VFG005767  VFG013327  VFG002027  VFG002480  VFG038916  VFG038839  VFG000907  VFG000344  VFG000344  VFG038916  VFG038722  VFG001386  VFG014950  VFG000344  VFG001089  VFG038839  VFG001206  VFG000926  VFG037064  VFG000431  VFG000332  VFG005286  VFG013070  VFG001386  VFG013070  VFG013070  VFG001311  VFG002440  VFG000754  VFG002180  VFG002480  VFG000841  VFG000390  VFG000841  VFG000119  VFG002180  VFG000907  VFG005776  VFG043465  VFG000925  VFG012509  VFG006672  VFG000130  VFG000344  VFG000078  VFG037118  VFG037064  VFG002186  VFG013065  VFG000173  VFG005776  VFG002176  VFG043350  VFG015000  VFG013248  VFG002176  VFG045332  VFG000812  VFG005776 | clpP  acpXL  katA  bopD  cap8D  cap8P  htpB  cpsA  msrA/B(pilB)  hasC  hasC  cpsJ  lplA1  hasC  cap8B  hasC  bauE  sodB  cpsJ  psaA  kdtB  cpsB  fabZ  luxS  cap8A  fbpA  phoP  cylG  cap8C  lspA  katA  relA  clpP  gtrB  msrA/B(pilB)  oatA  cpsG  phoP  msrA/B(pilB)  inhA  mucD  phoP  wbaP/rfbP  napA  phoP  hitC  tssH-5/clpV  fbpC  feoB  lirB  hitC  hitC  srtE  cylG  yhxB/manB  hldE  tssH-5/clpV  rtxB  flmH  hlyB  hitC  hitC  rtxB  AHA_1389  phoP  mucD  hitC  hpt  flmH  fbpC  fepD  mntB  tviB  rfaD  gbs0631  shuV  phoP  shuV  shuV  cap8O  bprB  bfpD  cpsK  tssH-5/clpV  hlyB  yscN  hlyB  algR  cpsK  hlyB  cylA  pfbA  fepC  iroC  fliI  algI  hitC  ami  recN  mntB  cpsE  shuT  phzS  cylA  cylB  fleN  mucP  msbA  cylB  llsG  escN  cylA | 82.6  68.1  67.2  66.1  63.3  61.1  56.9  56.9  56.8  55.4  54  52.2  52.1  51.9  51.7  51.4  49.8  49  48.4  46.4  45.3  45.1  43.5  43.4  42.2  41.7  41.6  41.3  41  40.5  40.3  40.2  39.8  39.3  39.3  39.1  39.1  39  39  38.2  37.6  37.5  37.1  36.6  36.5  36.3  35.9  35.9  35.4  35.3  35.1  35.1  35  35  34.9  34.9  34.6  34.5  33.9  33.8  33.6  33.5  33.5  33.3  33.3  33.1  32.9  32.8  32.8  32.7  32.6  32.3  32.2  32.2  32.2  32.2  31.9  31.7  31.7  31.4  31.4  31.2  31.1  31  30.9  30.7  30.7  30.4  30.3  30.3  30.1  30  29.5  29.4  29.2  29.2  28.9  28.6  28.5  28.3  28.2  28  27.9  27.8  27.8  27.7  27.6  27.5  27.4  27.4  27.3  27.1 | 7.96E-112  8.53E-26  3.42E-248  3.91E-159  7.53E-268  4.08E-168  2.09E-209  3.63E-99  3.43E-46  5.35E-47  1.45E-104  9.53E-82  1.07E-123  8.07E-100  3.74E-65  1.23E-16  6.06E-87  1.17E-67  8.89E-86  4.06E-100  7.26E-44  5.66E-70  1.20E-32  6.17E-37  2.39E-36  4.48E-140  1.29E-57  5.17E-60  9.26E-54  3.01E-31  2.51E-119  1.06E-185  2.07E-44  1.96E-71  1.09E-33  2.70E-134  1.73E-58  1.67E-51  3.25E-31  7.66E-65  3.99E-40  1.05E-52  3.76E-32  5.49E-26  5.14E-39  3.11E-43  2.84E-160  3.21E-61  1.37E-121  2.43E-32  1.71E-41  6.14E-46  4.57E-47  6.60E-39  3.81E-83  4.79E-14  3.05E-161  5.28E-88  5.55E-35  8.57E-27  8.48E-42  1.67E-55  2.95E-28  4.01E-47  6.88E-33  7.33E-46  4.02E-53  3.26E-69  1.36E-31  1.32E-29  5.07E-42  9.05E-37  2.80E-53  3.00E-28  4.03E-32  4.42E-44  3.84E-35  6.90E-26  7.73E-25  5.53E-53  4.58E-30  3.45E-26  1.68E-41  1.64E-101  9.54E-27  1.03E-20  1.25E-21  1.13E-27  1.59E-42  7.82E-25  1.05E-29  2.39E-20  4.17E-27  1.56E-57  2.01E-36  3.95E-22  7.61E-30  1.12E-55  4.11E-67  1.35E-20  5.13E-26  7.48E-20  4.82E-19  5.27E-31  1.77E-19  5.31E-14  4.63E-17  5.14E-55  4.17E-19  3.06E-08  1.61E-37  4.78E-21 | Stress protein  acyl carrier protein  Stress protein  Biofilm formation  Antiphagocytosis  Antiphagocytosis  Adherence  Antiphagocytosis  Stress protein  Antiphagocytosis; Adherence; Tissue invasion  Antiphagocytosis; Adherence; Tissue invasion  Antiphagocytosis  Intracellular growth  Antiphagocytosis; Adherence; Tissue invasion  Antiphagocytosis  Antiphagocytosis; Adherence; Tissue invasion  Iron uptake; Siderophore  Stress protein  Antiphagocytosis  Manganese uptake; ABC transporter  Endotoxin; Low toxicity; Adherence  Antiphagocytosis  (3R)-hydroxymyristoyl ACP dehydratase  S-ribosylhomocysteinase  Antiphagocytosis  Adherence; Chaperone  Regulation  3-ketoacyl-ACP-reductase CylG  Antiphagocytosis  Peptidase  Stress protein  Regulation  Stress protein  Endotoxin  Stress protein  Immune evasion  Antiphagocytosis  Regulation  Stress protein  Enzyme; Metalloprotease  serine protease MucD precursor  Regulation  undecaprenyl-phosphate galactosephosphotransferase  Proinflammatory effect  Regulation  Iron uptake; ABC transporter  Secretion system; Type VI secretion system  Iron uptake; ABC transporter  Iron uptake; Ferrous iron uptake  Dot/Icm type IV secretion system effector LirB  Iron uptake; ABC transporter  Iron uptake; ABC transporter  fimbrial associated sortase-  3-ketoacyl-ACP-reductase CylG  phosphomannomutase  Adherence; Phase variation; Structural mimicry  Secretion system; Type VI secretion system  RTX toxin transporter  short chain dehydrogenase/reductase family oxidoreductase  Toxin; Membrane-damaging; Pore-forming; RTX toxin  Iron uptake; ABC transporter  Iron uptake; ABC transporter  RTX toxin transporter  CobQ/CobB/MinD/ParA family protein  Regulation  serine protease MucD precursor  Iron uptake; ABC transporter  Intracellular growth; Cellular metabolism  short chain dehydrogenase/reductase family oxidoreductase  Iron uptake; ABC transporter  Iron uptake; Siderophore  Stress protein  Immune evasion  Endotoxin  Adherence; Sortase-assembled pili  Iron uptake; Heme uptake  Regulation  Iron uptake; Heme uptake  Iron uptake; Heme uptake  Antiphagocytosis  Secretion system; Type III secretion system; Invasion  Adherence; Type IV pilus  Antiphagocytosis  Secretion system; Type VI secretion system  Toxin; Membrane-damaging; Pore-forming; RTX toxin  Secretion system; Type III secretion system  Toxin; Membrane-damaging; Pore-forming; RTX toxin  Antiphagocytosis; Serum resistance  Antiphagocytosis  Toxin; Membrane-damaging; Pore-forming; RTX toxin  ABC (ATP-binding cassette) transporter CylA  Adherence; MSCRAMMs; Fibronectin-binding protein  Iron uptake; Siderophore  ATP binding cassette transporter  flagellum-specific ATP synthase FliI  Antiphagocytosis; Serum resistance  Iron uptake; ABC transporter  Adherence  Stress protein  Stress protein  Antiphagocytosis  Iron uptake; Heme uptake  Pigment; Antimicrobial activity  ABC (ATP-binding cassette) transporter CylA  Toxin; Hemolysin/bacteriocin; Biofilm formation  flagellar synthesis regulator FleN  metalloprotease protease  lipid transporter ATP-binding/permease  Toxin; Hemolysin/bacteriocin; Biofilm formation  Toxin; Pore-forming  Secretion system; Type III secretion system  ABC (ATP-binding cassette) transporter CylA |

Hits were retained for downstream interpretation using protein identity >27% and E-value <1e-5.
