# Supplementary material for: First fatal human bloodstream infection caused by Macrococcus caseolyticus subsp. caseolyticus in China: genomic insights into virulence and antimicrobial resistance
Source: Front Cell Infect Microbiol. 2026 Jun 1;16:1825695. doi: 10.3389/fcimb.2026.1825695 (PMC13265326; doi:10.3389/fcimb.2026.1825695)
Supplement: Supplementary file 4 [file Table4.docx]

| **Species Name** | **Strain** | **Accession Number** | **Role** |
| --- | --- | --- | --- |
| *Macrococcus caseolyticus* subsp. *caseolyticus* | WH712 | JBBEYG000000000 | Target Strain |
| *Macrococcus caseolyticus* subsp. *caseolyticus* | CCM 3540 | PZJF01000010 | Type Strain |
| *Macrococcus caseolyticus* subsp. *hominis* | CCM 7927 | CM010623 | Type Strain |
| *Macrococcus epidermidis* | Epi3002-OL | CP073819 | Reference |
| *Macrococcus goetzii* | CCM 4927 | CM010624 | Type Strain |
| *Macrococcus bohemicus* | 19Msa422 | CP054482 | Type Strain |
| *Macrococcus canis* | B8 | CP091281 | Reference |
| *Macrococcus armenti* | JEK29 | CP083602 | Reference |
| *Macrococcus bovicus* | LI0213 | CP128470 | Reference |
| *Macrococcus brunensis* | 18KM571 | CP092179 | Type Strain |
| *Macrococcus carouselicus* | ATCC 51828 | SCWD01000010 | Type Strain |
| *Macrococcus hajekii* | CCM 4809 | BMCC01000001 | Type Strain |
| *Macrococcus lamae* | CCM 4815 | SCWB01000010 | Type Strain |
| *Macrococcus equipercicus* | Epi0143-OL | CP073809 | Reference |
| *Staphylococcus aureus* subsp. *aureus* | NCTC 8325 | CP000253 | Outgroup |

**Table S3**. Genomes used for genus-level phylogenetic and ANI analyses of strain WH712.
