# Supplementary material for: First fatal human bloodstream infection caused by Macrococcus caseolyticus subsp. caseolyticus in China: genomic insights into virulence and antimicrobial resistance
Source: Front Cell Infect Microbiol. 2026 Jun 1;16:1825695. doi: 10.3389/fcimb.2026.1825695 (PMC13265326; doi:10.3389/fcimb.2026.1825695)
Supplement: Supplementary file 5 [file Table5.docx]

| Species Name | Strain | Accession Number | Role |
| --- | --- | --- | --- |
| *Macrococcus caseolyticus* subsp. *caseolyticus* | WH712 | JBBEYG000000000 | Target Strain |
| *Macrococcus caseolyticus* subsp. *caseolyticus* | JCSC5402 | GCF 000010585.1 | Reference |
| *Macrococcus caseolyticus* subsp. *caseolyticus* | FDAARGOS_868 | GCF 016028795.1 | Reference |
| *Macrococcus caseolyticus* subsp. *caseolyticus* | FDAARGOS_1005 | GCF 016127075.1 | Reference |
| *Macrococcus caseolyticus* subsp. *caseolyticus* | IMD0819 | GCF 002119825.1 | Reference |
| *Macrococcus caseolyticus* subsp. *caseolyticus* | 19Msa198 | GCF 024205865.1 | Reference |
| *Macrococcus caseolyticus* subsp. *caseolyticus* | 19Msa0287 | GCF 019357515.1 | Reference |
| *Macrococcus caseolyticus* subsp. *caseolyticus* | 19Msa0687 | GCF 019357555.1 | Reference |

**Table S4**. Genomes used for intra-subspecies Panaroo-based core-genome analysis of strain WH712.
